# Investigating the effects of table movement and sag on optical surrogate‐driven respiratory‐guided computed tomography

**DOI:** 10.1002/acm2.14565

**Published:** 2024-11-29

**Authors:** Niklas Lackner, Lou Dietrich, Andre Karius, Rainer Fietkau, Christoph Bert, Juliane Szkitsak

**Affiliations:** ^1^ Department of Radiation Oncology, Universitätsklinikum Erlangen, Friedrich‐Alexander‐Universität Erlangen‐Nürnberg Universitätsstraße 27 Erlangen Germany; ^2^ Comprehensive Cancer Center Erlangen‐EMN (CCC ER‐EMN) Erlangen Germany

**Keywords:** motion artifacts, respiratory‐guided CT, respiratory motion, surrogate system, table sag

## Abstract

**Purpose:**

Respiratory‐guided computed tomography (CT) typically employs breathing motion surrogates to feed image reconstruction or visual breathing coaching. Our study aimed to assess the impact of table movements and table sag on the breathing curves recorded in four‐dimensional (4D) CT and deep‐inspiration breath‐hold (DIBH) CT.

**Methods:**

For breathing curve measurements, static and dynamic phantom scenarios were used. Breathing curves were recorded using three different surrogate systems and the impact of table sag due to weights of up to 130 kg was analyzed and compared to a non‐weighted setting, respectively. The calibration procedure of the system used as an input for the visual coaching device used for clinical DIBH CT scans was adapted. We evaluated corresponding breathing curves acquired during DIBH and 4DCT scans of altogether 70 patients using various stability metrics.

**Results:**

The various surrogate systems showed consistent table sag measurements below 4 mm, even under loads up to 130 kg, compared to a reference scan conducted without additional weight. Higher weight loads were related to steeper table sag fall‐offs towards the deepest table position. For DIBH CT scans, the visual guidance was heavily affected by artifacts. This resulted in breathing threshold limits, which could not be achieved by 48% (*n* = 21) of the, respectively, examined patients. Using the new calibration workflow, the baseline drift was compensated better and 90% (*n* = 20) of the addressed patients stayed within the thresholds. The evaluated table sag in clinical 4DCT scans (*n* = 29) stayed below 3 mm compared to the non‐weighted situation.

**Conclusion:**

Table movement and sag can impact breathing curves recorded by different surrogate systems. Correcting table movement and sag artifacts is crucial for reliable breathing curve acquisition in respiratory‐guided CT.

## 1 INTRODUCTION

In the realm of respiratory‐guided computed tomography (CT) in medical imaging, four‐dimensional CT (4DCT), and deep‐inspiration breath‐hold (DIBH) CT have emerged as pivotal techniques.^1^ This holds especially in the context of thoracic and abdominal imaging, where respiratory motion can significantly affect image quality and diagnostic accuracy.^2^, ^3^, ^4^ With 4DCT, a three‐dimensional CT scan is created separately for several motion phases of a moving object, resulting in a dynamic dataset particularly useful for radiotherapy treatment planning.^5^ DIBH utilizes deep inspiration to increase the distance between the heart and chest, reducing radiation exposure to the heart and minimizing respiratory motion during imaging and therapy.^6^, ^7^

Central to the success of respiratory‐guided CT are surrogate systems, which have evolved to track respiratory motion with high precision. These systems are crucial for synchronizing imaging with the patient's breathing cycle in 4DCT. They are essential in DIBH to ensure a reproducible and reliable level of inhalation. For instance, corresponding optical tracking systems utilize infrared light to track targets attached with active or passive markers, and create a 3D representation based on the 2D data recorded by at least two respective charged‐coupled device (CCD) cameras. For a considerable period, such systems are a well‐established tool for surface‐guided radiation therapy (SGRT) and imaging in radiotherapy.^8^ Complementing these systems are visual guidance and feedback systems that provide real‐time feedback to patients during both DIBH and free‐breathing, ensuring consistent and accurate imaging throughout the procedure.^5^

However, an often‐overlooked aspect of respiratory‐guided CT is the impact of CT table sag and movement on the recorded breathing curve quality and hence image quality. Previous studies have highlighted the impact of table sag on capturing breathing signals in 4DCT,^9^, ^10^ indicating that table sag significantly influences recorded signals. Artifact‐affected breathing signals are supposed to lead to image artifacts, particularly in amplitude‐based image reconstruction.

The goal of the present study was thus to provide a comprehensive understanding of the consequences of CT table sag and table movement for the usage of different optical surrogate systems in respiratory‐guided CT. First, we explored the problem of table movement and sag for 4DCT and DIBH scans in phantom examinations. Second, we delved into the corresponding effects on clinical acquisitions, particularly focusing on our employed visual coaching device in DIBH scans. We also demonstrated an approach to counteract the issues of sag and movement through an adapted calibration technique and a two target tracking approach.

## 2 MATERIAL AND METHODS

### 2.1 Respiratory‐guided scanning

In this study, we used a Siemens SOMATOM go.Open Pro multi‐slice CT scanner (Siemens Healthineers AG, Forchheim, Germany) for both 3D DIBH CT and breathing‐adapted 4DCT^11^ scans. The 3DCT scans were acquired in helical scan mode with 0.5 s gantry rotation time, a pitch of 0.8, and 120 kV tube voltage. 4DCT scans were acquired in the sequence scanning mode with 120 kV tube voltage, 64 × 0.6 mm collimation, and a table increment of 0.9 × 64 × 0.6 mm. In 4D scans, rotation time is individually adapted to the breathing frequency of the patient by the intelligent 4DCT (Direct i4D) algorithm.^11^

### 2.2 Surrogate systems

**FIGURE 1:**
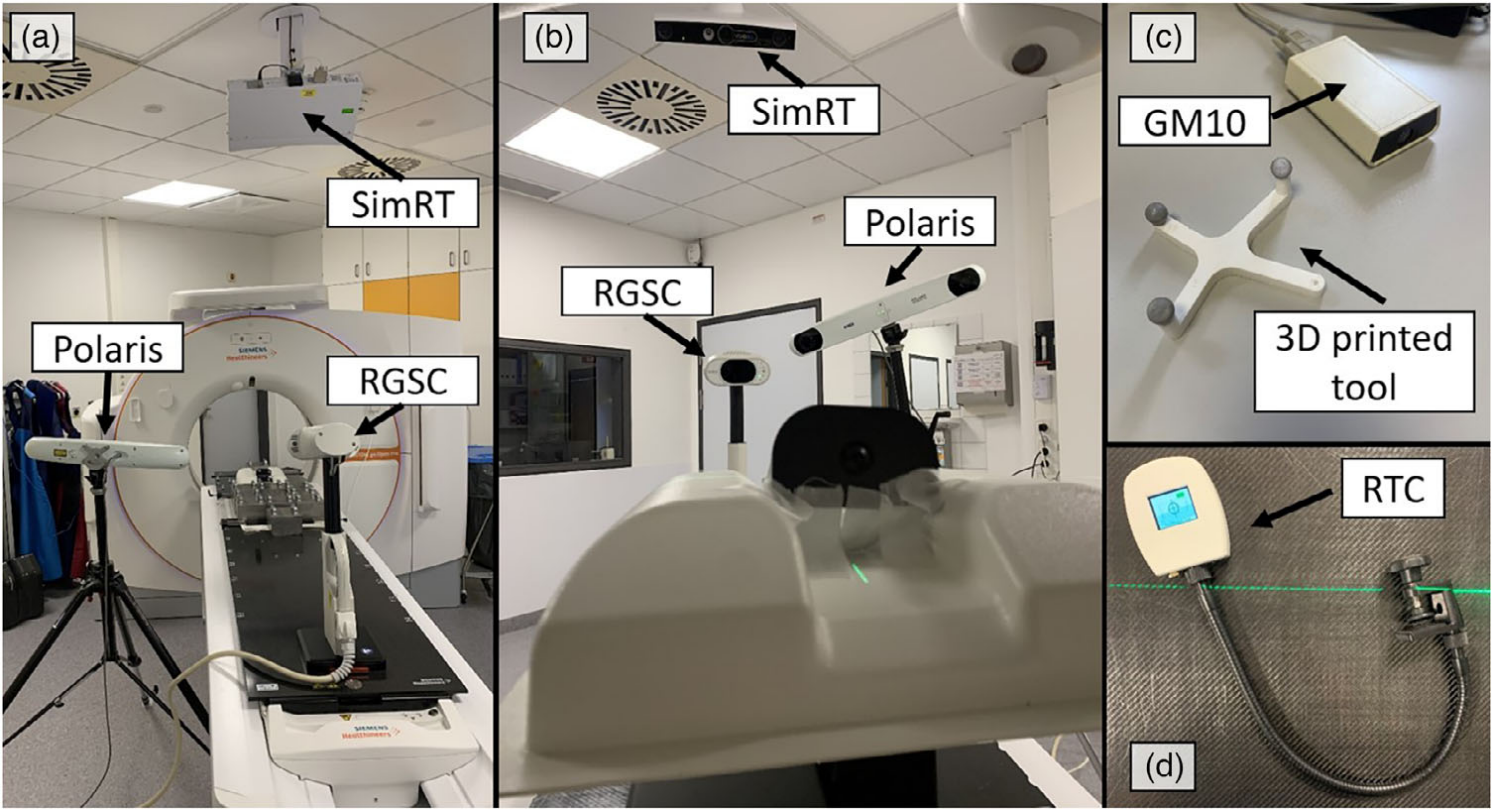
Measurement setup for the different surrogate systems. (a) Experimental setup view from camerasite. (b) The phantom's point of view showing all three surrogate systems. (c) Geiger Müller Counter to measure radiation and the 3D printed tool, which is used to track table movement. (d) Visual coaching device by VisionRT—Real Time Coach.

We evaluated three surrogate systems: Respiratory Gating for Scanner (RGSC; version 1.1.25.0, Varian Medical Systems, Inc. Palo Alto, California, USA), SimRT (version 7.2, VisionRT, London, UK), and a Polaris Spectra (Northern Digital Inc., Waterloo, Canada). The RGSC (Figure 1a,b) is a table‐mounted camera and uses infrared light reflections from a passive reflector block to determine patient breathing.

SimRT (Figure 1a,b) focuses on monitoring the patient‘s surface and uses a ceiling‐mounted pod with two sensors and a projector, which emits speckle patterns over a 20 × 50 cm^2^ body area. Position variations are then calculated by means of active stereo photogrammetry and triangulation within a 5 × 5 cm^2^ patch, which can be positioned individually anywhere on the captured patient's surface. The real‐time coach (RTC), an add‐on available for SimRT, is a detachable tool designed for visual coaching (Figure 1d), which is used in our clinical DIBH workflow to aid patients in breathing. This tool is specifically intended for a use for DIBH techniques, but should currently not be employed during the actual scanning process according to manufacturer recommendations. In our clinic, currently, we do not employ visual feedback throughout 4DCT scans.

In this study, the Polaris (Figure 1a,b) was used in combination with a tripod and coupled to a Geiger Müller Counter GM10 (Black Cat Systems, Westminster, Maryland, USA; Figure 1c), to temporally match the Polaris tracking information to an X‐ray signal. The Polaris excels in tracking multiple points with 0.06 mm precision (root mean square error) and about 16.6 ± 1 ms latency.^12^ The Polaris is a position sensor designed to determine the location of both active and passive infrared markers. Similar to the aforementioned camera systems, it employs the principles of stereo photogrammetry, which involves triangulating marker points using multiple calibrated views of a scene. These views are captured by two sensors embedded within the device, enabling accurate three‐dimensional localizations. We used NDI's 6D Architect (v3.02.04; Northern Digital Inc., Waterloo, Canada) for tool definition, so that the system was able to track both, the RGSC reflector block and a custom 3D printed object (Figure 1c) with infrared markers, that needed to meet the tool definition requirements of having at least three markers in a specified geometry. The Polaris Spectra was added to this study as an independent reference.

### 2.3 Light interference testing

Before and during the capture of breathing curves, the different surrogate systems were tested for signal loss to ensure the reliability and accuracy of data acquisition across RGSC, Polaris, and SimRT. This is crucial due to their sensitivity to precise lighting conditions, which could potentially cause cross‐system light interference affecting detection capabilities.

### 2.4 Initial calibration and adapted calibration procedure for SimRT

In SimRT, the surrogate's isocenter is set by placing a calibration plate at the CT scanner's isocenter. The breathing pattern is then measured from this point by tracking the position changes of a surface area (patch) using a rigid‐body transformation between the reference surface and its current state.^10^

**FIGURE 2:**
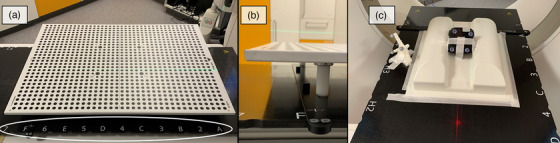
(a) Calibration setup with VisionRT's calibration plate. The plate is placed off‐center to visualize the table positions, which are highlighted by the white ellipse. (b) Close up view of how the calibration plate is fixated on a table position. (c) The static phantom measurement setup used for calibration evaluation.

For the initial calibration, the calibration plate is aligned at a fixed CT table position F using a fixation rail (Figure 2a,b). SimRT captures this position to confirm the calibration. An additional capture exactly 250 mm deeper in the bore helps to maintain an accurate breathing pattern detection, even if the table moves.

Unlike the RGSC system, which is mounted on the table, SimRT does not move with the table. Therefore, it requires specific correction values for table movement. These values ensure that the patch used for breathing information can be tracked along despite a moving table. In this study, the correction values in SimRT software were set as follows: 64 slices, 0.6 mm collimation, 0.8 pitch, 0.5 s rotation time (resulting in a table velocity of 87.77 mm/s), 25 mm ramp‐up distance for helical 3DCT scans, and 34.5 mm table increment for axial 4DCT scans. These corrections apply as soon as the system receives an X‐ray‐on signal from the CT scanner. Due to the table's ramp‐up movement before the X‐ray acquisition and thus the corrections start, the patch jumps, causing artifacts that appear as peaks in the breathing curve.

To evaluate the impact of different calibration plate positions on the breathing signal recorded with SimRT, we positioned the plate at table positions 5, 6, and 7 (as shown in Figure 2a,b). Static phantom measurements were conducted with the phantom being consistently placed at the location depicted in Figure 2c. This allowed us to assess the breathing signals resulting from each calibration. Patch positioning for all SimRT measurements was fixed at table position E. We utilized a 3D DIBH scan protocol with a scan length of 32.9 cm starting at the identical CT table position.

After a first learning period using the initial calibration, we adapted the calibration procedure by positioning the calibration plate at the deepest CT position 5. Unless otherwise specified, all breathing signals recorded with SimRT in this study used the adapted calibration.

### 2.5 Static phantom measurements

**FIGURE 3:**
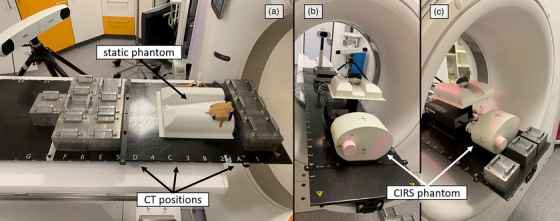
Experimental setup for (a) static measurements with the static phantom here depicted with 130 kg added. Dynamic phantom setup with the CIRS phantom in (b) a non‐weighted and (c) a weighted scenario. In the dynamic phantom setup, the table marker is positioned at the same table position as the RGSC reflector block.

As static phantom, we utilized a VisionRT quality assurance phantom called “thorax test phantom” with a weight of roughly 500 g and placed it in a typical patient position. The phantom was fixated to the table using tape to ensure stability. The RGSC reflector block was placed on top of the phantom, the 3D printed tool was placed on the table at the same table position. Both were fixated using tape (Figure 3a). The weights utilized in this study are solid blocks of lead, each weighing approximately 13 kg. Linear regression analysis was performed to examine the correlation between weight and table sag for each system, where the independent variable was weight, and the dependent variables were table sag from each system. Model parameters were estimated using the ordinary least squares (OLS) method. A mixed linear model (MLM) approach was employed to evaluate sag variations among these systems, with weight considered as a continuous predictor. Shapiro‐Wilk tests were performed to assess the normality of the sag data for each system. Since the *p*‐values were greater than 0.05, indicating normal distribution, independent two‐sided *t*‐tests were used to evaluate the statistical significance of the differences between systems.

We conducted static phantom measurements in two scenarios: one without weights and another with incremental weights added up to 130 kg at the table's head end. Next, we assessed how the phantom's position affected sag across seven different positions by shifting the table longitudinally within the gantry, while varying the loads each time. Weight was again added at the table head end, and the RGSC reflector block was moved along table positions 1 to 4. The longitudinal measurement was only evaluated using the Polaris camera, due to the field of view restrictions of the other cameras. The DIBH CT scans for this setup were done with a fixed scan length, always starting from the same position. Total table sag was calculated using the breathing signal in a stable region, averaging the signal between 9 and 10 s after the first X‐ray‐on.

Additionally, we conducted a measurement without adding extra weight. During this measurement, the SimRT system was recalibrated with its calibration plate set to position F to ensure alignment of the calibration plate's center with the patch position. This ensured an accurate assessment of table sag under non‐weighted condition.

### 2.6 Dynamic phantom measurements

For dynamic measurements, we employed the dynamic thorax motion phantom model 008A [Computerized Imaging Reference Systems (CIRS) Norfolk, Virginia, USA]. This model is designed to replicate the size and shape of a human thorax. It is constructed from materials that mimic the densities of human tissue, bone, and lung, and roughly weighs 17.2 kg.^13^ To render its motion detectable by our surrogate systems, the thorax test phantom with the RGSC reflector block attached to it, was positioned atop the surrogate platform of the CIRS phantom, as depicted in Figure 3b. Additionally, the 3D printed tool was placed on the table at the same position as the RGSC reflector block, allowing the Polaris system to track table sag and movements. This setup enabled the creation of a phantom signal corrected for both table motion and sag using the Polaris.

The dynamic thorax phantom was used to simulate two types of respiratory motions: Regular breathing motion to simulate patient breathing during 4DCT scans and regular breathing followed by a DIBH pattern. Correspondingly, image acquisitions involved both 4DCT and 3DCT DIBH scans. All scans were again conducted using varying weight conditions under differing table movement scenarios (either static or dynamic table). The extra weight was first added at the end of the table (position 1) and then also in front of the phantom starting at table position 5 (Figure 3a).

### 2.7 4DCT patient measurements

Patient measurements were performed to confirm the phantom results in a clinical setup. The 4DCT patient cohort included 29 patients with tumors in the lungs, liver, and kidney. Among the 25 patients (16 males, 9 females) with available demographic data (for details see supplementary material, Table A), the mean age was 66 ± 14 years and the mean weight was 69 ± 13 kg. Of these, 19 had lung tumors, and 6 had upper abdominal tumors. Breathing curves were acquired using our clinical camera systems RGSC and SimRT. Each patient was positioned on the CT table, and the RGSC reflector block was positioned on the abdominothoracic patient region. The tracking point for SimRT was set and deliberately chosen to be located directly below the RGSC reflector block to guarantee similar input motion. Respiratory data were extracted for the systems. Table sag was determined for each patient and surrogate system. For comparison, curves were temporally matched to the first X‐ray‐on signal.

### 2.8 DIBH patient measurements

The positive impact on patient breathing due to breathing training using visual feedback was already demonstrated in other studies.^14^, ^15^ In this study, we focused on the effect of table sag and table movement on the breathing data of DIBH patients potentially impacting the visual coaching. We investigated the reproducibility and stability of the DIBH technique using visual coaching across a cohort of 41 patients with left‐sided breast cancer (for details see supplementary material, Table B). The cohort was split into two groups: Cohort‐1 (*n* = 21) used the old calibration, while Cohort‐2 (*n* = 20) used the new adapted calibration. Cohort‐1 had a mean weight of 74 ± 14 kg and a mean age of 51 ± 12 years, while Cohort‐2 had a mean weight of 69 ± 12 kg and a mean age of 59 ± 14 years. Patient age and weight were not available for one patient in Cohort‐1 and three patients in Cohort‐2. Similar to the 4DCT scans, each patient was placed on the CT table but without the reflector block, as we used only SimRT to capture breathing information.

At first, a breathing training trainA outside of the CT bore was done prior to moving the patient into the scan position, where a second training session trainB was performed and finally the scan was executed in DIBH. During the training procedure, the patients were instructed to engage in normal breathing for approximately half a minute, followed by a 8 to 15‐s DIBH, with clear start and stop signals. This procedure was repeated three times each, with visual feedback. To ensure consistent breathing patterns across different DIBHs and individuals, patients were asked to focus on chest breathing rather than abdominal breathing. Visual coaching used the RTC, connected via Bluetooth to the SimRT workstation, to show the patients their respiratory signals as well as a pre‐set DIBH target window with a 1.5 mm threshold.

### 2.9 DIBH metrics

**FIGURE 4:**
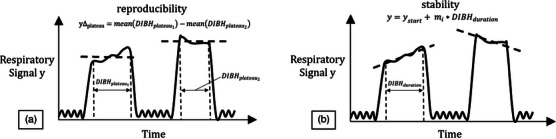
Graphical representation of (a) reproducibility and (b) stability in DIBH. Figure adapted from^14^.

The concept of reproducibility and stability in DIBH scans has been explored in literature among others by Cervino et al.^14^ and Stock et al.^15^ For our study, we chose a similar approach and we used

(1) $$y\Delta_{\mathrm{train}}=\text{mean}\left({\text{DIBH}}_{\mathrm{trainA}}\right)-\text{mean}\left({\text{DIBH}}_{\mathrm{trainB}}\right)$$

(2) $$y\Delta_{\text{X-ray}}=\text{mean}\left({\text{DIBH}}_{\mathrm{trainB}}\right)-\text{mean}\left({\text{DIBH}}_{\text{X-ray}}\right)$$

as our metrics for the reproducibility, where ${\text{DIBH}}_{\mathrm{trainA}}$ represents the breathing amplitude during the first coached DIBH plateau, ${\text{DIBH}}_{\mathrm{trainB}}$ the second coached plateau right before the scan, and ${\text{DIBH}}_{\text{X-ray}}$ the plateau achieved during the scan, as depicted in Figure 4a. $y\Delta_{\mathrm{train}}$ and $y\Delta_{\text{X-ray}}$ are expressed in millimeters and a low value indicates good reproducibility. Stability, on the other hand, is defined as the maximum change in amplitude among the DIBHs in each series, calculated between the start and end points of each DIBH. This change is determined by fitting the data with a line starting from the start of the DIBH plateau until the end of the plateau

(3) $$y=y_{\text{start}}+m_{i}*{\text{DIBH}}_{\text{duration}},$$

where $y_{\text{start}}$ is the start of the DIBH plateau, $m_{i}$ represents the slope (or the drift) of the linear fit for each DIBH (measured in mm/DIBH) and ${\text{DIBH}}_{duration}$ is the full DIBH as shown in Figure 4b. A low value of $m_{i}$ indicates good stability and little drift of the DIBH signal. We evaluated the drifts $m_{\mathrm{trainA}}$, $m_{\mathrm{trainB}}$, and $m_{\text{X-ray}}$ of the breath‐holds ${\text{DIBH}}_{\mathrm{trainA}}$, ${\text{DIBH}}_{\mathrm{trainB}}$ and ${\text{DIBH}}_{\text{X-ray}}$. We conducted Shapiro‐Wilk tests to assess normality, which indicated that several metrics were not normally distributed (*p* < 0.05). Subsequently, we performed independent two‐sided Wilcoxon rank‐sum tests across the different reproducibility and stability metrics of the cohorts. Additionally, we evaluated how many patients were able to stay within the 1.5 mm threshold. In total, being less than 1 s outside the threshold was counted as a pass.

### 2.10 Data analysis

Data analysis was conducted using MATLAB (version R2019b; The MathWorks Inc., Massachusetts, USA). The static and dynamic phantom breathing curves were exported from the different surrogate systems. The effects of added weight were examined, and the resulting baseline shift due to table sag was evaluated. Baseline shift in a breathing curve refers to a deviation from the normal baseline level of respiration. Table sag (or baseline shift) was evaluated in phantom but also in patient 4DCT acquisitions by calculating the difference of the average of the first two signal minimum values compared to the average of the last two minimum values (minimum values corresponding to the negative breathing valleys). For the dynamic DIBH phantom acquisitions, the drift was calculated using the stability metrics from Section 2.9. In general, the X‐ray signals from the different systems were used to temporally match breathing curves.

## 3 RESULTS

### 3.1 Light interference tests

Before and during the SimRT, RGSC, and Polaris were actively collecting data for different DIBH and 4DCT phantom scans, we visually evaluated the tracking effectiveness of the different systems. No scan showed any loss of tracking for the different camera systems.

### 3.2 Impact of calibration plate position on SimRT breathing signal

A strong impact on the measured breathing signal for different calibrations was shown. We found an increase of breathing signal by 0.6 ± 0.1 mm (0.02 mm increase per cm scanned) for calibration in position 5, a drop in breathing signal of −0.6 ± 0.1 mm (0.02 mm drop per cm scanned) for a calibration in position 6, a drop in breathing signal by −1.2 ± 0.1 mm (0.04 mm drop per cm scanned) for a calibration in position 7; the initial calibration at position F showed a drop in breathing signal by −1.4 ± 0.1 mm (0.04 mm drop per cm scanned). The calibrations on different positions lead to different breathing sag, due to the impact of table sag and the different corrective patch propagations. For all subsequent measurements presented in this study, unless otherwise mentioned, the calibration plate was set at position 5.

### 3.3 Static phantom measurements

**FIGURE 5:**
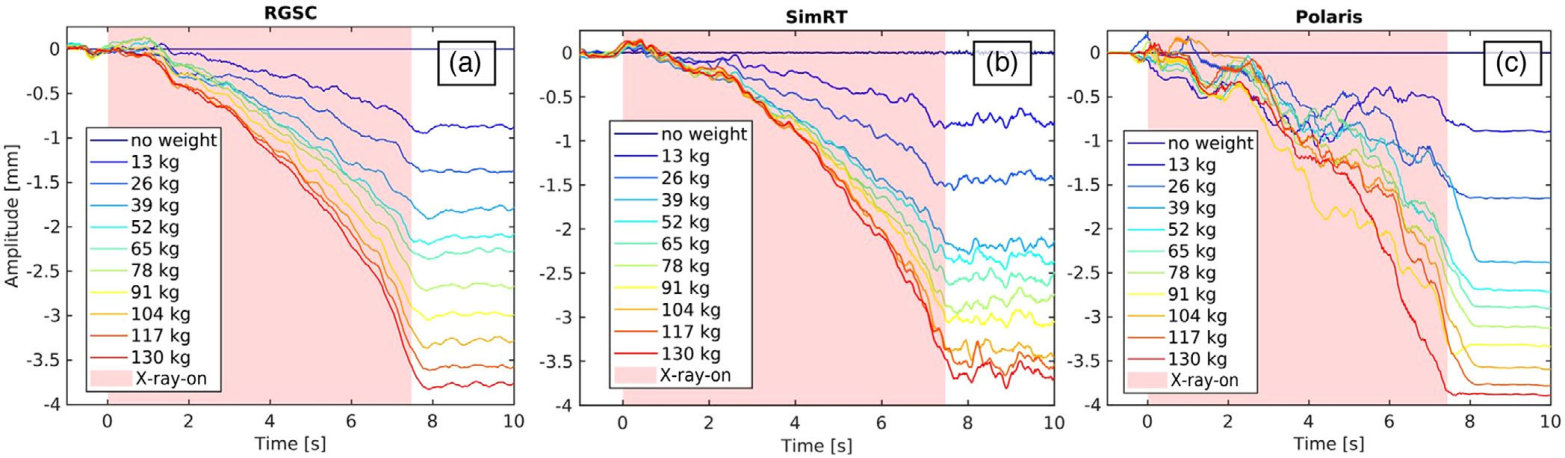
Table sag response to varying loads ranging from a non‐weighted scenario up to an additional weight of 130 kg for different surrogate systems: (a) RGSC, (b) SimRT, and (c) Polaris.

In this section, we present the static phantom measurement results for table sag using three different camera systems: RGSC, SimRT, and Polaris. The analysis compared the systems' response to incremental loading and its effect on table sag. The investigation into table sag across the RGSC, SimRT, and Polaris camera systems revealed a consistent pattern of behavior as the load on the table increased. Starting from a non‐weighted scenario and progressing up to a total weight of 130 kg, each system exhibited a sag response indicating a degree of linearity under load. Figure 5 offers a clear visual depiction of how the RGSC, SimRT, and Polaris camera systems respond to varying weights placed on the table. The colored lines represent change in table sag when subjected to weights from 0 to 130 kg, tracked over time. The “X‐ray‐on” sector is particularly noteworthy as it marks the region where the table experiences movement and increased bending.

For the non‐weighted scenario SimRT measured −1.41 ± 0.09 mm while RGSC measured a sag of −1.39 ± 0.03 mm. The results in Table 1 detail the degree of sag observed at each weight increment for all three systems, compared to their non‐weighted situation to make them comparable. These measurements progressively increase with weight, highlighting the incremental deformation of the table surface. The linear regression analysis revealed a strong, statistically significant correlation between weight and table sag measurements for all systems. For each kilogram increase in weight, the table sag decreased by approximately 0.023 mm (Polaris, *R* ^2^ = 0.914), 0.024 mm (RGSC, *R* ^2^ = 0.992), and 0.023 mm (SimRT, *R* ^2^ = 0.936). These high *R*‐squared values indicate a good fit for the linear models. Application of MLMs showed a statistically significant negative correlation between weight and sag measurements for all systems (*p* < 0.001). Sag differences between RGSC, SimRT, and Polaris were not significant according to the independent two‐sided *t*‐tests (RGSC vs. Polaris, *p* = 0.26; SimRT vs. Polaris, *p* = 0.60; RGSC vs. SimRT, *p* = 0.89). The longitudinal sag profile further shows how this sag was distributed across different table positions, from positions 1 through 4, with distinct values at 52 , 78 , and 104 kg weights.

**TABLE 1:** Table sag for different optical surrogate systems in dependency of weight and longitudinal table sag profile in dependency of position and weight.

Table sag (weight) Compared to non‐weighted				Longitudinal table sag—Polaris (position, weight) Compared to non‐weighted			
Weight (kg)	Polaris (mm)	RGSC (mm)	SimRT (mm)	Table position	52 kg (mm)	78 kg (mm)	104 kg (mm)
*13*	*−0.90 ± 0.01*	*−0.86 ± 0.03*	*−0.78 ± 0.07*	*1*	−3.61 ± 0.01	−4.25 ± 0.01	**−4.93 ± 0.01**
*26*	*−1.64 ± 0.01*	*−1.34 ± 0.02*	*−1.46 ± 0.07*	*A*	−3.14 ± 0.01	−3.67 ± 0.01	**−4.25 ± 0.01**
*39*	*−2.38 ± 0.01*	*−1.75 ± 0.02*	*−2.18 ± 0.15*	*2*	−2.64 ± 0.01	−3.10 ± 0.01	**−3.59 ± 0.01**
*52*	*−2.70 ± 0.01*	*−2.09 ± 0.03*	*−2.36 ± 0.08*	*B*	−2.39 ± 0.01	−2.66 ± 0.01	**−2.99 ± 0.01**
*65*	*−2.90 ± 0.01*	*−2.25 ± 0.03*	*−2.55 ± 0.09*	*3*	−1.89 ± 0.01	−2.01 ± 0.01	**−2.18 ± 0.01**
*78*	*−3.12 ± 0.01*	*−2.63 ± 0.02*	*−2.83 ± 0.09*	*C*	−1.35 ± 0.01	−1.34 ± 0.01	**−1.50 ± 0.01**
*91*	*−3.33 ± 0.01*	*−2.93 ± 0.02*	*−3.08 ± 0.07*	*4*	−1.09 ± 0.01	−0.95 ± 0.01	**−0.88 ± 0.01**
*104*	*−3.59 ± 0.01*	*−3.23 ± 0.02*	*−3.44 ± 0.09*				
*117*	*−3.78 ± 0.01*	*−3.53 ± 0.02*	*−3.51 ± 0.13*				
***130***	***−3.89 ± 0.01***	***−3.76 ± 0.02***	***−3.67 ± 0.08***				

Scenarios with the highest sag are highlighted in bold.

### 3.4 Dynamic phantom measurements – 4DCT

**FIGURE 6:**
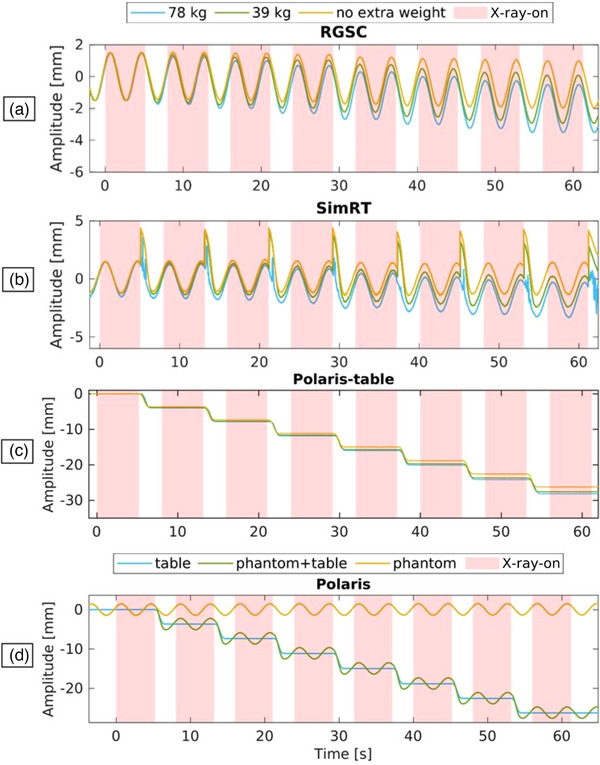
Response of the different surrogate systems for the weighted and the situation with no weight in a 4DCT acquisition. (a) RGSC breathing signal, (b) SimRT breathing signal, (c) Polaris table signal tracking the 3D printed tool, (d) Polaris breathing signal in green, Polaris table signal in blue, Polaris table movement corrected signal in yellow for the 4DCT acquisition.

The provided plots in Figure 6 show the amplitude of respiratory signals against time under varying loads during 4DCT acquisitions. The RGSC breathing signal (Figure 6a) exhibits table sag of −0.5 ± 0.02 mm with no extra weight, −1.4 ± 0.02 mm with 39 kg extra weight, and −2.0 ± 0.03 mm with 78 kg extra weight. The SimRT breathing signal (Figure 6b) shows table sags of −0.08 ± 0.13 mm with no extra weight, increasing to −1.26 ± 0.15 mm with 39 kg, and −1.84 ± 0.12 mm with 78 kg. To correct for table motion between the X‐ray‐on states the SimRT system jumps the patch from one position to another depending on the table increment. This yields breathing curve peak artifacts between the switch from X‐ray‐on to X‐ray‐off, even for the last switch, where there is no table increment. For the Polaris system, table sags (Figure 6c) compared to the non‐weighted case are −1.32 ± 0.01 mm with 39 kg extra weight and −1.89 ± 0.01 mm with 78 kg extra weight. Figure 6d displays the Polaris system's tracking of a phantom's movement in relation to the table movement during 4DCT acquisitions. It effectively separates the phantom's respiratory signal from the table's positional signal, allowing for the deduction of the table's influence.

### 3.5 Dynamic phantom measurements—DIBH

**FIGURE 7:**
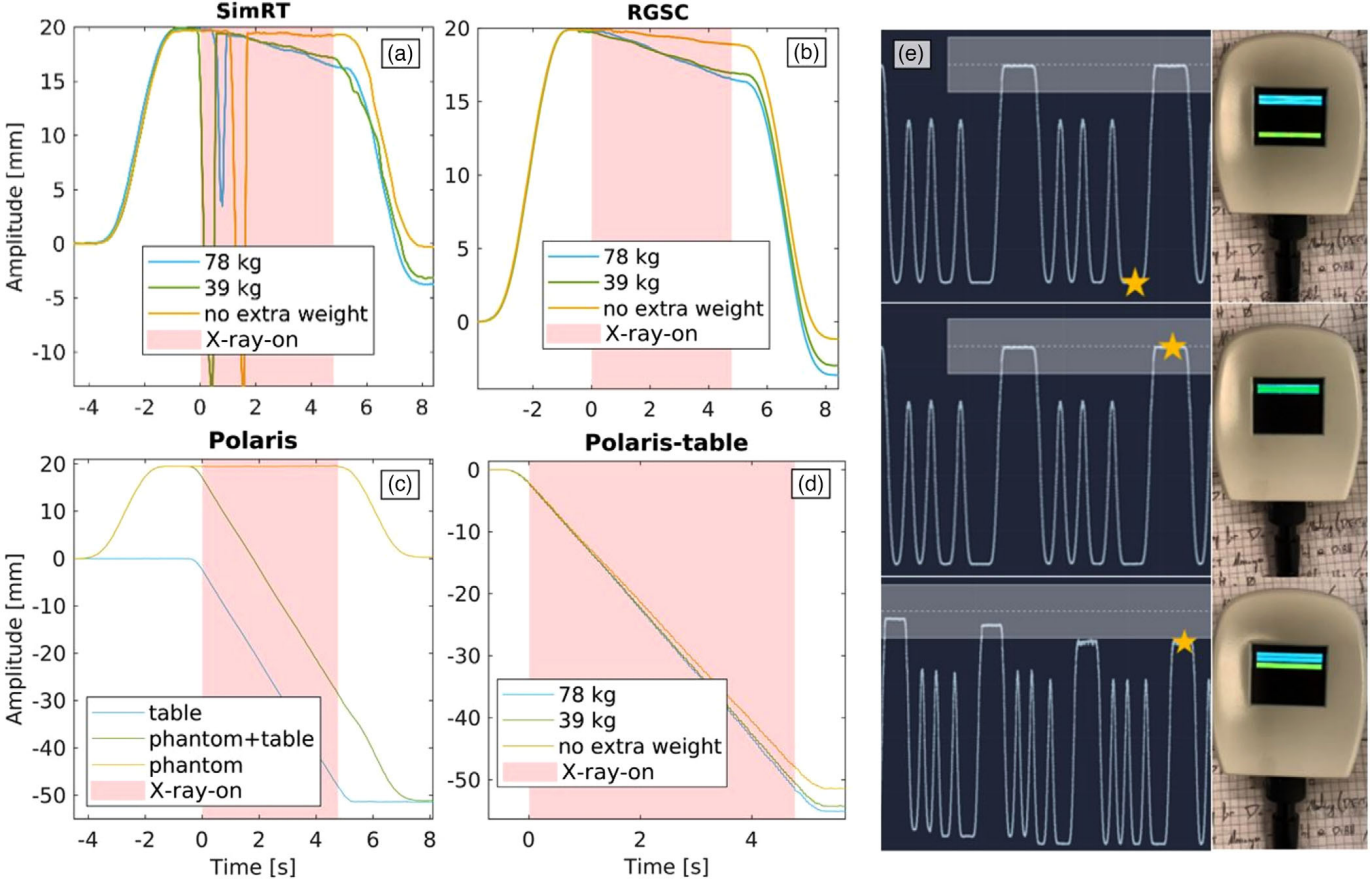
Response of the different surrogate systems for the weighted and the situation with no weight in a DIBH acquisition. (a) SimRT breathing signal, (b) RGSC breathing signal, (c) Polaris breathing signal in orange, Polaris table signal in blue, Polaris table movement corrected signal in yellow, (d) Polaris table signal tracking the 3D printed tool, (e) top and middle images show the threshold set in SimRT (white on the left graph and in blue on the RTC) for the non‐weighted situation and the bottom image shows weight being added between breath‐holds and its impact on the RTC. The yellow star indicates the breathing state, which the RTC displays.

For the dynamic DIBH phantom measurements, it was observed that for the RGSC system, the table sag measurements were −1.1 ± 0.02 mm with no extra weight, −2.98 ± 0.03 mm with an additional 39 kg, and −3.63 ± 0.02 mm with an extra 78 kg (Figure 7b). For the SimRT system, the table sag was −0.2 ± 0.09 mm with no extra weight, −3.11 ± 0.08 mm with 39 kg extra, and −3.75 ± 0.08 mm with 78 kg extra weight (Figure 7a). For the Polaris system, while the table sag with no extra weight was used to compare against, it was found to be −3.02 ± 0.01 mm with 39 kg extra weight, and −3.81 ± 0.01 mm with 78 kg extra weight (Figure 7d). Just as for the 4DCT acquisitions, the Polaris effectively separates the phantom's respiratory signal from the table's positional signal, allowing for the deduction of the table's influence. The result is a corrected breathing signal that accounts for and removes the table movement (Figure 7c). Figure 7e shows the direct impact of weight on the RTC and the visual coaching.

### 3.6 4DCT patient measurements

**FIGURE 8:**
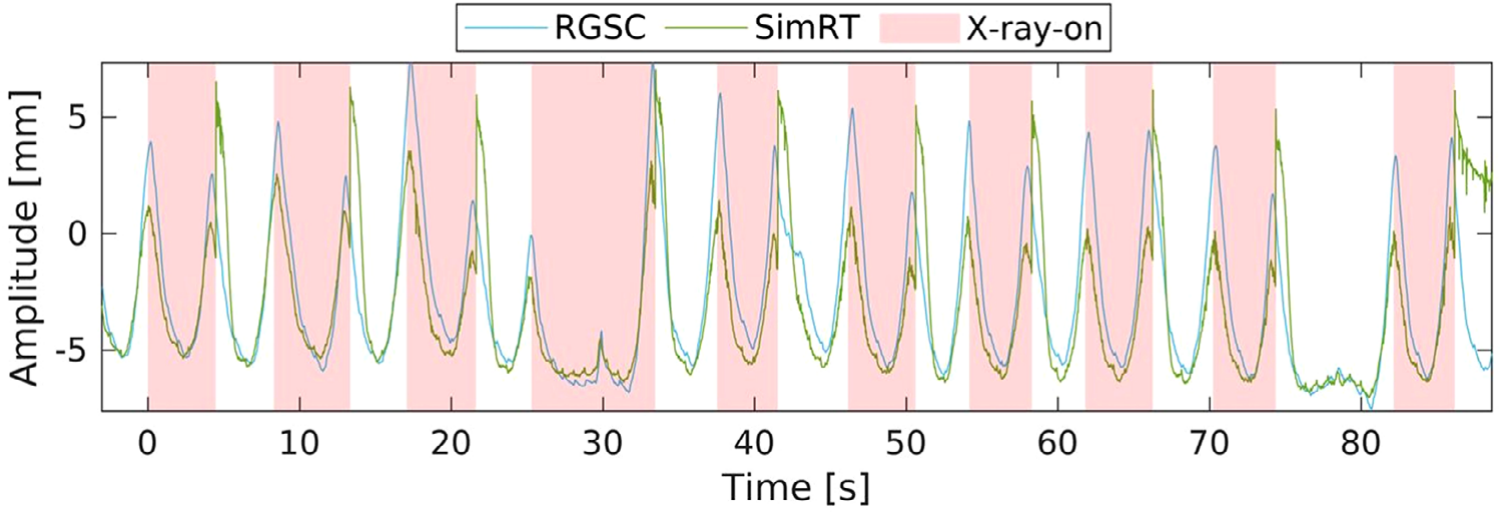
Illustration of a typical patient's (83 years, 76.8 kg) curve obtained by the two camera systems RGSC and SimRT in a breathing‐adapted axial 4DCT scan.

In our analysis of 29 clinical 4DCT scans, the general form of the curves across all 29 patients appeared consistent (Figure 8), displaying a good qualitative match between the systems in both phase and amplitude. Similar to the results from phantom measurements, it is important to note that the SimRT system's ceiling‐mounted configuration, as opposed to the table‐mounted RGSC setup, necessitates correction for table movement. The vendor‐specific correction is implemented during the transition from X‐ray‐on to X‐ray‐off, leading to observable peak artifacts, as illustrated in Figure 8. We observed the following outcomes related to the baseline sag of patient curves: RGSC showed an average baseline sag of −1.74 ± 0.69 mm, whereas the SimRT recorded an average of −1.38 ± 0.66 mm. The number of table increments averaged 9.20 ± 1.06 and the total distance measured averaged 317.40 ± 36.51 mm. The observed differences between the breathing amplitudes can be attributed to the varying placement of surrogate markers, RGSC derives its signal from the movement of the reflector block, whereas the SimRT's patch is positioned slightly below this block and derives its information directly from the patient surface.

### 3.7 DIBH patient measurements

This study evaluated the reproducibility and stability of DIBH techniques across two patient cohorts using SimRT accompanied by a visual coaching device the RTC. Cohort‐1 was measured with the old calibration and Cohort‐2 was measured using the adapted calibration method.

**FIGURE 9:**
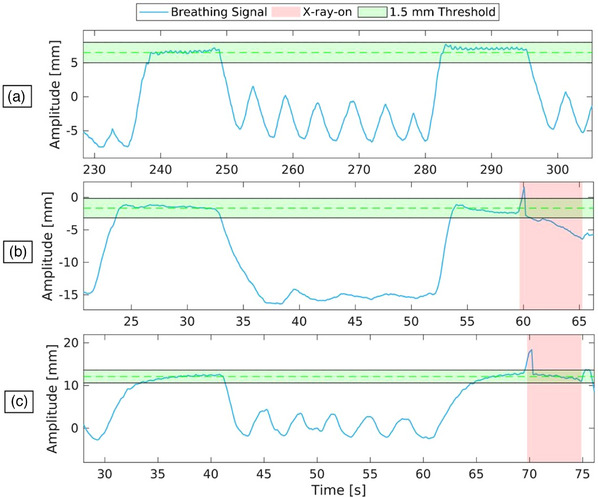
Representation of three different breathing curves associated with DIBH scans. (a) Representative breathing curve generated from DIBH training, captured before the actual scanning of a patient (36 years, 64 kg). (b) Representative DIBH breathing curve from a patient (36 years, 64 kg) in Cohort‐1. (c) Representative breathing curve from a Cohort‐2 patient (59 years, 61 kg).

When assessing the reproducibility of the breath‐hold during training ($y\Delta_{\mathrm{train}}$) and actual scan scenarios ($y\Delta_{\text{X-ray}}$), both cohorts exhibited similar levels of reproducibility. In Cohort‐1, $y\Delta_{\mathrm{train}}$ was 0.48 ± 0.68 mm, and in Cohort‐2, it was 0.78 ± 1.24 mm (see Figure 9a). For the reproducibility during the actual scan scenarios ($y\Delta_{\text{X-ray}}$) for Cohort‐1, the value was 0.20 ± 1.83 mm, and for Cohort‐2, it was 0.33 ± 1.00 mm (refer to Figure 9b).

Additionally, we analyzed the stability of the breath‐hold, focusing on potential drift in the signals. In Cohort‐1, the first training session ($m_{\mathrm{trainA}}$) showed a drift of 0.19 ± 1.76 mm/DIBH, and the second session ($m_{\mathrm{trainB}}$) had a drift of −0.02 ± 1.31 mm/DIBH. The actual scan ($m_{\text{X-ray}}$ revealed a drift of −5.33 ± 3.68 mm/DIBH (Figure 9, graph in the middle). For Cohort‐2, the first training session ($m_{\mathrm{trainA}}$) indicated a drift of −0.54 ± 1.51 mm/DIBH, and the second session ($m_{\mathrm{trainB}}$) showed a drift of 0.06 ± 1.19 mm/DIBH. Notably, Cohort‐1 exhibited a significantly larger drift during the actual scan ($m_{\text{X-ray}}$: −5.33 ± 3.68 mm/DIBH) compared to Cohort indicating a potential greater instability in breath‐hold during actual scans in Cohort‐1. During the actual scan ($m_{\text{X-ray}}$), the drift was −2.09 ± 2.04 mm/DIBH (Figure 9c). These results are summarized in Table 2.

**TABLE 2:** Summary of reproducibility and stability outcomes across the two distinct patient cohorts.

	$y\Delta_{\mathrm{train}}$ (mm)	$y\Delta_{\text{X-ray}}$ (mm)	$m_{\mathrm{trainA}}$ (mm / DIBH)	$m_{\mathrm{trainB}}$ (mm / DIBH)	$m_{\text{X-ray}}$ (mm / DIBH)
*Cohort‐1 (n = 21)*	*0.48 ± 0.68*	*0.20 ± 1.83*	*0.19 ± 1.76*	*−0.02 ± 1.31*	***−5.33 ± 3.68***
*Cohort‐2 (n = 20)*	*0.78 ± 1.24*	*0.33 ± 1.00*	*−0.54 ± 1.51*	*0.06 ± 1.19*	***−2.09 ± 2.04***

Results significantly different (*p* < 0.05) between the two cohorts are highlighted in bold.

The independent two‐sided Wilcoxon rank‐sum tests indicate no significant difference (*p* > 0.05) in $y\Delta_{\mathrm{train}}$(*p* = 0.5), $y\Delta_{\text{X-ray}}$ (*p* = 0.81), $m_{\mathrm{trainA}}$(*p* = 0.24), and $m_{\mathrm{trainB}}$ (*p* = 0.79) between the cohorts. However, there is a statistically significant difference (*p* < 0.05) in the $m_{\text{X-ray}}$(*p* = 5.62 × 10^−6^) dataset. Looking at the clinical thresholds set in practice for Cohort‐1, 48% of patients drifted out of the set threshold limits for longer than 1 s, and for Cohort‐2, 90% of patients could stay within threshold limits.

## 4 DISCUSSION

This discussion dives into how CT table movement and sag affect the reliability of different optical systems for guiding respiratory CT scans. We started by examining how table movement and sag impact both 4DCT and DIBH scans in tests with phantoms. Then, we explore how these factors affect real clinical scans, focusing on the effectiveness of our visual coaching tool. We also demonstrated our method to tackle these challenges, using a modified calibration technique and a dual‐target tracking approach.

The phenomenon of table sag or movement during CT scans is a variable aspect that depends significantly on the specific CT scanner and the table being used. Each model of CT scanner and its accompanying table may exhibit unique characteristics in terms of stability and movement dynamics. Manufacturers design these systems with certain tolerances for movements, but factors like mechanical wear, patient weight, and the design of the moving parts can lead to differences in how much and in what manner the table sags or moves during a scan.^16^ Inherent sag in CT tables, worsened by patient weight, often leads to a sag in recorded patient breathing patterns.^17^ However, it is crucial to note that any table sag due to CT table movement would not be reflected in most treatment linear accelerator setups. During commissioning, the movement of the table should be checked in the vertical direction when loaded with weights simulating an average adult patient.^18^

Optical surrogate systems that are not fixed to the CT table need to account for table motion and sag, while systems fixed to the table need to tackle table sag, to ensure accurate respiratory signals. In the worst‐case scenario, this could necessitate patient re‐scanning, leading to an unintended additional radiation dose.^19^ The use of visual coaching devices that rely on surrogate systems may result in the introduction of artifacts, which could lead to inaccurate breathing information and potentially cause confusion among patients.

In static phantom measurements we compared table sag under increasing loads using RGSC, SimRT, and Polaris. Key findings revealed that all three systems exhibited a similar response to increased weight, with minor variations in measured sag between the systems at different weight increments, which were non‐significant. The different behavior observed in Polaris data for the region, where motion occurred can be attributed to the methodology used for calculating table sag and the inherent uncertainties during the measurement process. Table sag was normalized using a non‐weighted baseline. This normalization and higher uncertainties during movement (X‐ray‐on region) of the markers can lead to varying results, resulting in a noisier signal. In contrast, when the table is in a static position (before and after the X‐ray region), the measurements are more reliable and align with the expected linear relationship as weights increased.

For the dynamic phantom measurements, we distinguished between DIBH and 4DCT measurements. Overall, the Polaris effectively separated the phantom's respiratory signal from the table's positional signal using a multi‐tool tracking approach, allowing for the deduction of the table's influence. The result was a corrected breathing signal that accounted for the table movement and sag, providing a clearer representation of the phantom's actual respiratory pattern under different loading conditions.

One notable limitation of this study is the positioning of the phantoms. While efforts were made to approximate typical patient scenarios, the placement of the CIRS surrogate platform which was notably off‐axis in relation to the examination table. This discrepancy in positioning could potentially lead to variations when comparing the study results to real‐life patient cases.

In the clinical DIBH setting, both considered cohorts demonstrated similar levels of reproducibility, but differences in stability particularly during actual scans became evident. These findings suggest that while reproducibility was consistently achieved, it might be necessary to address the stability challenges in DIBH application. Further research into the factors influencing stability, such as better control and separation of table sag and table movement, could enhance the efficacy of DIBH protocols.

For the clinical 4DCT scans, we found consistent curve forms across all 29 scans, with RGSC and SimRT showing qualitative matches. However, the ceiling‐mounted SimRT's correction for table movement introduces peak artifacts, which could pose issues for breathing‐adapted 4DCT. These correction peaks might interfere with the X‐ray trigger selection,^19^ or cause issues in the reconstruction. This can potentially lead to artifacts or confuse the imaging algorithm. Additionally, the total sag observed in our study raised concerns for conventional 4DCT scanning. When table sag is not accounted for, modern 4DCT algorithms can compensate for it.^20^ However, conventional algorithms, especially those using amplitude‐based reconstruction, may experience inaccuracies.^21^, ^22^ It also has to be noted that in the 4DCT patient group the reflector block and SimRT patch were positioned similarly, but not identically. Furthermore, both considered systems have distinct methodologies for the estimation of vertical breathing amplitudes, and further investigations for proving their equivalence for breathing‐adapted 4DCT are required.

Our findings emphasize the need for careful consideration of system‐specific characteristics and calibration methods in clinical 4DCT and DIBH CT scans to ensure accuracy in the imaging procedure. The influence of patch placement on amplitude and phase dependency, in relation to human physiology, presents a fascinating area for future research.^23^

For the DIBH scans, the employed adapted calibration method can be viewed as an unconventional workaround that deviates from the standard usage recommended by the manufacturer. Nevertheless, the adapted workflow, which simulates a deeper patch position, enhanced the stability of DIBH scans. However, there is a notable issue: the ramp‐up movement introduces a spike‐like artifact in the DIBH signal. SimRT can currently not address this behavior because its correction algorithms begin only after the first X‐ray‐on signal, which occurs after the table has already reached a certain velocity during the ramp‐up phase. Despite the achieved improvements, about 10% of patients still struggle to maintain proper breath‐hold (with deviations less than 1 s to set threshold). It is therefore advisable to turn off visual feedback during a DIBH scan. We suggest this course of action because concerns such as table sag or movement have the potential to distort visual feedback. This distortion could inadvertently lead to the patient adjusting their breathing based on inaccurate information, which could impact the effectiveness of their treatment. Breathing during the scan would be absolutely undesirable as it could introduce motion artifacts, potentially necessitating a rescan. Alternatively, it is possible to tell the patient to ignore visual feedback during the actual scan, so that breath‐hold data is still captured for retrospective evaluation. Instead of relying solely on the adapted calibration approach, it would be advantageous if the vendor's current calibration and daily quality assurance system were to include compensation for the sag induced by patient weight on the table. This enhancement would contribute to a more comprehensive and efficient quality assurance process. Another approach would be to address table sag during calibration by adjusting weight distribution, which could mitigate sag effects during movements. However, this may introduce additional stress to the workflow due to increased complexity.

To highlight the importance of the table sag issue, a recently published study^24^ explored the importance of calibrating the RGSC system to enhance accuracy and stability. It noted that the current method for calibrating a wall‐mounted RGSC system often resulted in a considerable residual error. The study concluded that employing a three‐block calibration method can effectively mitigate table sag due to table movement in DIBH and 4DCT scans for the wall‐mounted RGSC system.

A potential solution regarding table movement and sag artifacts may involve transferring table movement data from the scanner to surrogate systems, which could address issues related to table movement. However, this does not completely resolve the issue of table sag. Note that in the present study, our investigation was limited to visual feedback during and prior to DIBH scans only. However, it would be beneficial to explore two additional aspects: (a) implementing visual coaching for 4DCT patients, given that many artifacts in 4DCT are attributed to irregular breathing patterns, even in modern 4DCT approaches and coaching may stabilize patient breathing; and (b) directly addressing table movement and sag issues during DIBH scans. This would not only coach patients during the scanning process through visual feedback but also ensure the collection of more reliable data for evaluation.

## 5 CONCLUSION

We found severe table movement and sag artifacts for breathing curves using various surrogate systems in respiratory‐guided CT. In several patient cases, the visual breathing coaching signals drifted out of the set threshold limits. Adaptation of the calibration workflow led to better compensation of the baseline drift and thus visual coaching becoming more stable. Correction for table movement artifacts is considered crucial when using respiratory curves for respiratory‐guided CT.

## AUTHOR CONTRIBUTIONS

All author contributed significantly to the performed work and approved the final version of the manuscript to be published. Conceptualization: Niklas Lackner, Lou Dietrich, Andre Karius, Christoph Bert, Juliane Szkitsak. Methodology: Niklas Lackner, Andre Karius, Christoph Bert, Juliane Szkitsak. Software/Tools Development: Niklas Lackner. Data collection: Niklas Lackner, Lou Dietrich. Data analysis: Niklas Lackner. Writing—Original Draft Preparation: Niklas Lackner. Writing—Review & Editing: Niklas Lackner, Lou Dietrich, Andre Karius, Christoph Bert, Juliane Szkitsak, Rainer Fietkau. Supervision: Andre Karius, Christoph Bert, Juliane Szkitsak.

## CONFLICT OF INTEREST STATEMENT

The Universitätsklinikum Erlangen and the Department of Radiation Oncology have institutional research grants with Siemens Healthineers AG and VisionRT that are not directly related to the present work.

## Supporting information



Supporting Information

## Data Availability

Data are provided by the corresponding author upon reasonable request.
