# Supplementary material for: Investigating the effects of table movement and sag on optical surrogate‐driven respiratory‐guided computed tomography
Source: J Appl Clin Med Phys. 2024 Nov 29;26(2):e14565. doi: 10.1002/acm2.14565 (PMC11799900; doi:10.1002/acm2.14565)
Supplement: Supplementary file 1 — Supporting Information [file ACM2-26-e14565-s001.docx]

***Table A.*** *Demographic data available for the 25 4DCT patients including age, weight and cancer site.*

| ***4DCT*** | *Age (years)* | *Weight (kg)* | *Cancer site* |  |
| --- | --- | --- | --- | --- |
| *Pat01* | 54 | 67 | *Lung metastasis* |  |
| *Pat02* | 58 | 64 | *Adrenal Gland* |  |
| *Pat03* | 58 | 73 | *Lung metastasis* |  |
| *Pat04* | 89 | 67 | *Lung* |  |
| *Pat05* | 52 | 56 | *Lung* |  |
| *Pat06* | 100 | 62 | *Lung metastasis* |  |
| *Pat07* | 71 | 55 | *Liver* |  |
| *Pat08* | 41 | 67 | *Lung* |  |
| *Pat09* | 75 | 82 | *Lung* |  |
| *Pat10* | 58 | 82 | *Liver* |  |
| *Pat11* | 61 | 77 | *Liver* |  |
| *Pat12* | 61 | 67 | *Lung* |  |
| *Pat13* | 76 | 79 | *Lung* |  |
| *Pat14* | 84 | 80 | *Lung* |  |
| *Pat15* | 71 | 55 | *Liver* |  |
| *Pat16* | 51 | 90 | *Lung* |  |
| *Pat17* | 86 | 69 | *Lung* |  |
| *Pat18* | 56 | 42 | *Lung* |  |
| *Pat19* | 83 | 76 | *Lung* |  |
| *Pat20* | 73 | 59 | *Lung* |  |
| *Pat21* | 58 | 63 | *Lung* |  |
| *Pat22* | 56 | 52 | *Lung* |  |
| *Pat23* | 64 | 59 | *Abdomen* |  |
| *Pat24* | 68 | 97 | *Lung* |  |
| *Pat25* | 51 | 90 | *Lung* |  |

| ***Cohort-1*** | *Weight (kg)* | *Age (years)* |  | ***Cohort-2*** | *Weight (kg)* | *Age (years)* |
| --- | --- | --- | --- | --- | --- | --- |
| *Pat01* | 73 | 45 |  | *Pat01* | 46 | 53 |
| *Pat02* | 72 | 44 |  | *Pat02* | 75 | 61 |
| *Pat03* | 62 | 72 |  | *Pat03* | 66 | 80 |
| *Pat04* | 61 | 55 |  | *Pat04* | not available | 48 |
| *Pat05* | 75 | 76 |  | *Pat05* | 67 | 35 |
| *Pat06* | 71 | 58 |  | *Pat06* | 58 | 50 |
| *Pat07* | 72 | 44 |  | *Pat07* | 61 | 59 |
| *Pat08* | 118 | 65 |  | *Pat08* | not available | 44 |
| *Pat09* | 90 | 43 |  | *Pat09* | 43 | 64 |
| *Pat10* | 73 | 45 |  | *Pat10* | 93 | 83 |
| *Pat11* | 85 | 54 |  | *Pat11* | 79 | 64 |
| *Pat12* | 64 | 36 |  | *Pat12* | 73 | 44 |
| *Pat13* | 64 | 49 |  | *Pat13* | 64 | 40 |
| *Pat14* | 64 | 36 |  | *Pat14* | 69 | 66 |
| *Pat15* | 64 | 36 |  | *Pat15* | 77 | 48 |
| *Pat16* | 61 | 41 |  | *Pat16* | 79 | 79 |
| *Pat17* | 95 | 67 |  | *Pat17* | 70 | 66 |
| *Pat18* | 82 | 64 |  | *Pat18* | 79 | 64 |
| *Pat19* | 80 | 63 |  | *Pat19* | 79 | 79 |
| *Pat20* | 58 | 40 |  |  |  |  |

***Table B.*** *Demographic data available for the 37 DIBH patients including age and weight. Data is split into Cohort-1 (n=20) and Cohort 2 (n=17).*
